# Supplementary material for: The Escherichia coli MFS-type transporter genes yhjE, ydiM, and yfcJ are required to produce an active bo3 quinol oxidase
Source: PLoS One. 2023 Oct 20;18(10):e0293015. doi: 10.1371/journal.pone.0293015 (PMC10588857; doi:10.1371/journal.pone.0293015)
Supplement: S1 Fig — (PDF) [file pone.0293015.s001.pdf]

YFcJ

```
CcoA      MT---QT---------------------AASAPPLPLRNLIVLVAAQAFLGAQMSMIFTV
YfcJ      MTAVSQTETRSSANFSLFRIAFAVFLTYMTVGLPLPVIPLFV--HHELGYGN--------
          **   **                      : . ***:  *:*    :   *

CcoA      GGLAGQSLATNPCLATLPLSLIVLGSVLTAQPMSSFMAVYGRRAGFILATAAGGI-GAAI
YfcJ      ------------TMVGIAVGIQFLATVLTRGYAGRLADQYGAKRSALQGMLACGLAGGAL
                       :. : :.: .*.:***    . :   ** : . : .  * *: *.*:

CcoA      S-AHAL---AIGSFPLFCLGSLLAGIYMSAQ--GFYRFAATDGIAPEHQSKAISWVLAGG
YfcJ      LLAAILPVSAPFKFALLVVGRLILGFGESQLLTGALTW-GLGIVGPKHSGKVMSWNGMAI
            *  *   *  .* *: :* *: *:  *    *   : . . :.*:*..*.:**   .

CcoA      LAAAVLGPQLVKLTAQALVVPFQATYLAIIAINLAGPLIFAFLRIPAPGRRVKGQAGGRT
YfcJ      YGALAVGAPLGLLI----------------------------------------------
           .* .:*  *  *

CcoA      RGELLRDPVILVAMICGMVSYALMNLVMTSTPLAVVGCGHTTTNAADIVSAHVLAMYLPS
YfcJ      ---------------HSHYGFAALAITTMVLPVLAWACNGTVRKVPALAG------ERPS
                          .  .:* : :.    *: . .*. *. :.  :..        **

CcoA      FFTGHLIARFGRETIVGIGLFILAVAGAVALTGVDLEQFFLALMLLGLGWNFGFIGSTAM
YfcJ      LWSV-----VGLIWKPGLGLALQG-------VGFAVIGTFVSLYFASKGWAMAGFT----
          :::      .*     *:** : .       .*. :   *::* : . ** :. :

CcoA      LAAAHAPEERGTVQGMNDFVVFGGVFLASLSSGGLMTC--------------ASADAVAG
YfcJ      ------------------LTAFGGAFVVMRVMFGWMPDRFGGVKVAIVSLLVETVGLLLL
                            :..***.*:.     * *                 :.. :

CcoA      WQAVNLAM---LPFLTLAGAALIWLVLR-------PKDTR--------------------
YfcJ      WQAPGAWVALAGAALTGAGCSLIFPALGVEVVKRVPSQVRGTALGGYAAFQDIALGVSGP
          *** .  :      ** **.:**: .*        *.:.*

CcoA      ------------------------------------
YfcJ      LAGMLATTFGYSSVFLAGAISAVLGIIVTILSFRRG
```

YhjX

```
CcoA            MTQTAASAPPLPLRNLIVLVAAQAFLGAQMSMIFTVGGLAGQSLATNPCLATLPLSLIVL
YhjX            MTPSNYQ--RTRWLTLIGTIITQFALGSVYTWSLFNGALSA-KLDAPVSQVAFSFGLLSL
                ** :  .       .**  : :*  **:  :  :  *.*:. .* :  . .:: :.*: *

CcoA            GSVLTAQPMSSFMAVYGRRAGFILATAAGGIGAAISAHALAIGSFPLFCLGSLLAGIYMS
YhjX            GLAISSSVAGKLQERFGVKRVTMASGILLGLGFFLTAHSDNL--MMLWLSAGVLVGLA--
                * .:::.  ..:   :* :   : :    *:*  ::**:  :  : *:  ..:*.*:

CcoA            AQGFYRFAATDGIAPEHQSKAISWVLA----------GGLAAAVLGPQLVKLTAQALVVP
YhjX            --------DGAGYL-LTLSNCVKWFPERKGLISAFAIGSYGLGSLGFKFIDTQL-LETVG
                           *      *:.:.*.            *. . . ** :::.      .*

CcoA            FQATYLAIIAINLAGPLIFAFLRIPAPGRRVKGQAGGRTR----GELLRDPVILVAMICG
YhjX            LEKTFVIWGAIALLMIVFGATLMKDAPKQEVKTSNGVVEKDYTLAESMRKPQYWMLA---
                :: *::   ** *   :: * *   ** :.** . *   :    .* :*.*   :

CcoA            MVSYALMNLVMTSTPLAVVGCGHTTTN---AADIVSA------HVLAMYLPSFFTGHLIA
YhjX            -----VMFLTACMSGLYVIGVAKDIAQSLAHLDVVSAANAVTVISIANLSGRLVLGILSD
                     :* *.   : * *:* .:  ::     *:***        :*     :. * *

CcoA            RFGRETIVGIGLFILAVAG-AVALTGVDLEQFFLALMLLGLGWNFGFIGSTAMLAAAHAP
YhjX            KIARIRVITIGQVISLVGMAALLFAPLNAVTFFAAIAC--VAFNFG--GTITVF-----P
                ::.*  :: ** .*  *.  *: :: ::   ** *:    :.:***  *: :::     *

CcoA            EERGTVQGMNDFVVFGGVFLASLSSGGLMTCASADAVAGWQAVNLAMLPFLTLAGAALIW
YhjX            SLVSEFFGLNNLAKNYGVIYLGFGIGSICGSIIASLFGGFYVTFYVIFALLIL-SLALST
                .  . . *:*::.   **:  .:. *.:  .  *. ..*: ..  .:: :* * . **

CcoA            LVLRPKDTR--------
YhjX            TIRQPEQKMLREAHGSL
                 : :*::.
```

YebQ

```
CcoA      MTQTAASAPPLPLRNLIVLVA---------------------------------------
YebQ      MPKVQADGLPLPQRYGAILTIVIGISMAVLDGAIANVALPTIATDLHATPASSIWVVNAY
          * :. *.. *** *   :*.

CcoA      ---------AQAFLGAQMSM--IFTVGGLAGQSLATNPCLATLPLSLIVLGSVLTAQPMS
YebQ      QIAIVISLLSFSFLGDMFGYRRIYKC-GLVVFLLSSLFCALSDSLQMLTLARVIQGFGGA
                   : :***  :.   *:.  **.   *::  *  :  *.::.*. *: .   :

CcoA      SFMA--------V-----YGRRAG---FILATAAGGIGAAISAHALAIGSFPLFCLGSLL
YebQ      ALMSVNTALIRLIYPQRFLGRGMGINSFIVA-VSSAAGPTIAAAILSIASWKWLFLINVP
          ::*:        :      **  *   **:* .:.. * :*:*  *:*.*:  : * .:

CcoA      AGIYMSAQGFYRFAATDGIAPEHQS--------KAISWVL-AGGLAAAVLGPQLVKLTAQ
YebQ      LGIIALLL-AMRFLPPNGSRASKPRFDLPSAVMNALTFGLLITALSGFAQGQSLTLIAAE
           **        **   :*   .:          :*::: *   .*:. . * .*. ::*:

CcoA      ALVVPFQATYLAIIAINLAGPLIFAFLRIPAPGRRVKGQAGGRTRGELLRDPVILVAMIC
YebQ      LVVMVVVGIFFI-----------RRQLSLPVPL----------LPVDLLRIPLFSLSICT
           :*: . . ::               * :*.*              :*** *:: :::

CcoA      GMVSYALMNLVMTSTPLAVVG-CGHTTTNAADIVSAHVLAMYLPSFFTGHLIARFGRETI
YebQ      SVCSFCAQMLAMVSLPFYLQTVLGRSEVETGLLLTPWPLATMVMAPLAGYLIERVHAGLL
          .: *:.   *.*.* *: :    *:: .::. :::   **  : : ::*:** *.    :

CcoA      VGIGLFILAVAGA--VAL--TGVDLEQFFLALMLLGLGWNFGFIGSTAM-LAAAHAPEE-
YebQ      GALGLFIMAAGLFSLVLLPASPADIN-IIWPMILCGAG--FGLFQSPNNHTIITSAPRER
           .:****:*..    * *  : .*:: ::  ::* * *  **:: *       : **.*

CcoA      RGTVQGMNDFVVFGGVFLASLSSGGLMTCASADAVAGWQA--VNLAMLPFLTLAGAALIW
YebQ      SGGASGMLGTA-----RLLGQSSGAALVALMLNQF-GDNGTHVSLMAAAILAVIAACVSG
           * ..** . .      * . ***. :..   : . * :.  *.*    :*:: .*.:

CcoA      L-VLRPKDTR
YebQ      LRITQPRSRA
        \: :*:.
```

YnfM

```
CcoA        MTQ--TAASAPPL-------PLRNLIVLVAAQAFLGAQM----------SMIFTVGG---
YnfM        MSRTTTVDGAPASDTDKQSISQPNQFIKRGTPQFMRVTLALFSAGLATFALLYCVQPILP
            *::  *. .**            * ::  .:  *: . :          :::: *

CcoA        LAGQSLATNPCLATLPLS----LIVLGSVLTAQPMSSFM--------------------A
YnfM        VLSQEFGLTPANSSISLSISTAMLAIGLLF-TGPLSDAIGRKPVMVTALLLASICTLLST
            : .*.:. .*. ::: **    ::.:* :: : *:*. :                    :

CcoA        VYGRRAGFILATAAGG--------IGAAISAHALAIGSFPLFCLGSLLAGIYMSAQGFYR
YnfM        MMTSWHGILIMRALIGLSLSGVAAVGMTYLSE-EIHPSFVAFSMG-----LYISGNSIGG
            :     *:::  *  *        :* :  :.     **  *.:*     :*:*.:.:

CcoA        FAATDGIAPEHQSKAISWVLAGGLAAAVLGPQLVKLTAQALVVPFQATYLAIIAINLAGP
YnfM        MS--GRLISGVFTDFFNWRIALA---------------------------AIGCFALASA
            ::  . :     :. :.* :* .                           ** .: **.

CcoA        LIFAFLRIPAPGRRVKGQ------------AGGRTRG--ELLRDPVILVAMICGMVSYAL
YnfM        LMF--WKILPESRHFRPTSLRPKTLFINFRLHWRDRGLPLLFAEGFLLMGSFVTLFNYIG
            *:*   :*   .*:.:                 * **   *: : .:*:. :  :..*

CcoA        MNLVMTSTPLAVVGCGHTTTNAADIVSAHVLAMYLPSFFTGHLIARFGRETIVGIGLFIL
YnfM        YRLML--SPWHV------SQAVVGLLSLAYLTGTWSSPKAGTMTTRYGRGPVMLFS----
             .*::  :*  *      :  ...::*   *:    *  :* : :*:**  :: :.

CcoA        AVAGAVALTGVDLE------QFFLALMLLGLGWNFGFIGSTAMLAAAHAP---EERGTVQ
YnfM        --TG-VMLFGLLMTLFSSLWLIFAGMLL----FSAGFFAAHSVASSWIGPRAKRAKGQAS
              :* * * *: :        :* .::*    :. **:.: :: ::  .*   . :* ..

CcoA        GMNDFVVFGGVFLASLSSGGLMTCASADAVAGWQAVNLAMLPFLTLAGAALIWLVLRPKD
YnfM        SLYLFSYYLGSSIAGTL-GGV-----FWHNYGWNGVGAFIALMLVI------ALLVGTRL
            .:  *  : *  :*.   **:          **:.*.  :  :*.:       *::  :

CcoA        TR----
YnfM        HRRLHA
             *
```

YdiM

```
CcoA        MTQTAASAPPLPLRNLIVLVAAQAFLGAQMSMIF--TVGGLAGQSLATNPCLATLPLSLI
YdiM        -----MKNPYFP---------------TALGLYFNYLVHG--------------------
                  . * :*               : :.: *   * *

CcoA        VLGSVLTAQPMSSFMAVYGRRAGFILATAAGGIGAAISAHALAIGSFPLF----------
YdiM        -MGVLLMSLNMASLETLWQ--------TNAAGV--SIVISSLGIGRLSVLLFAGLLSDRF
             :* :* :  *:*: :::         * *.*:  :*   :*.** : ::

CcoA        -----------CLGSLLAGIYMSAQG--FYRFAATDGIAP-------------------E
YdiM        GRRPFIMLGMCCYMAFFFGILQTNNIIIAYVFGFLAGMANSFLDAGTYPSLMEAFPRSPG
                       *  ::: **  : :    * *.   *:*

CcoA        HQSKAI-SWVLAGGLAAAVLGPQLVKLTAQALVVPFQATYLAIIAIN-LAGPLIFAFLRI
YdiM        TANILIKAFVSSGQF----LLPLIISLLVWA-ELWFGWSFMIAAGIMFINALFLYRCTFP
              .  * ::* :* :    * * ::.* . *  : *  :::   .*  : . :::

CcoA        PAPGRRVKGQAGGRTRGELLRDPVILVAMICGMVSYALMNLVMTSTPLA----VVGCGHT
YdiM        PHPGRRLPVIKKTTSSTEH-RCS------IIDLASYTLYGYISMATFYLVSQWLAQYGQF
            * ****:       :  *  *        * .:.**:* . :  :*       :.  *:

CcoA        TTNAADIVSAHVLAMY-----LPSFFTGHLIARFGRETIVGIGLFILAVAGAVALTGVDL
YdiM        VAGMSYTMSIKLLSIYTVGSLLCVFITAPLIRNTVRPTTL---LMLYTFISFIALFTVCL
            .:. :  :* ::*::*     *  *:*. ** .  * * :   *:: :. . :**  * *

CcoA        EQFFLALMLLGLGWNFGFIGS------TAMLAAAHAPEERGTVQGMNDFVVFGGVFLASL
YdiM        HPTFYVVIIF--AFVIGFTSAGGVVQIGLTLMAERFPYAKGKATGIYYSAGSIATFTIPL
            .  * .::::  .: :** .:         * * : *  :*.. *:   .   ..*   *

CcoA        SSGGLMTCASADAVAGWQAVNLAMLPFLTLAGAALIWLVLRPKD-TR------------
YdiM        ITAHLSQ--RSIADIMWFDTAIAAIGFLL-----ALFIGLRSRKKTRHHSLKENVAPGG
             :. *     : *   *  . :* : **       ::: ** :. **
```

YhjE

```
CcoA      MT------QTAASAPPLPLRNLIVLVAAQAFLGAQM-----------------SMIFTVG
YhjE      MQATATTLDHEQEYTPINSRNKVLVA---SLIGTAIEFFDFYIYATAAVIVFPHIFFPQG
          *       :   .  *:  ** :::.   :::*: :                  ::*  *

CcoA      GLAGQSLATNPCLATLPLSLIVLGSVLTAQPMSS-FMAVYGRR----AGFILATAAGGIG
YhjE      DPTAATL---QSLATFAI-------AFVARPIGSAVFGHFGDRVGRKATLVASLLTMGIS
          . :. :*    .***: :       .:.*:*:.* .:. :* *    * :: :  : **.

CcoA      AAISAHALAIGSFPLFCLGSLLAGIYMSA----Q-----GFY---RFAATDGIAPEH---
YhjE      ------TVVIGLLPGYATIGIFAPLLLALARFGQGLGLGGEWGGAALLATENAPPRKRAL
                ::.** :* :.  .::* : ::     *     * :    : **:.  *.:

CcoA      ------------------QSKAISWVLAGGLAAAVLGPQLVKLTAQALVVPFQATY-LAI
YhjE      YGSFPQLGAPIGFFFANGTFLLLSWLLTDEQFM-----------SWGWRVPFIFSAVLVI
                                :**:*:.               : .  ***  :  *.*

CcoA      IAI----NLAGPLIFAFLRIPAPGRRVKGQAGGRTRGELLRDPVIL--VAMICGMVSYAL
YhjE      IGLYVRVSLHESPVF--EKVAKAKKQVKIP-----LGTLLTKHVRVTVLGTFIMLATYTL
          *.:    .*    :*   ::    ::**        * ** . * :  :. :  :.:*:*

CcoA      MNLV----M-TSTPLAVVGCGHTTTNAADIVSAHVLAMYLPSFFTGHLIARFGRETIVGI
YhjE      FYIMTVYSMTFSTAAAPVGLGLPRNEVLWMLMMAVIGFGVMVPVAGLLADAFGRRKSMVI
          : ::    *  **  * ** *   .:.  ::   *:.: :   .:* *   ***.. : *

CcoA      G-L--FILAVAGAVALTGVDLEQFFLALMLLG---LGWNFGFIGSTAMLAAAHAPEERGT
YhjE      ITTLIILFALFAFNPLLGSGNPILVFAFLLLGLSLMGLTFGPMGALL---PELFPTEVRY
               :::*: .   * * .   :.:*::***   :* .** :*:         * *

CcoA      VQGMNDFVVFGGVFLASLSSGGLMTCASADAVAGWQAVNLAML----PFLTLAGAALIWL
YhjE      T---------GASFS--YNVASILGASVAPYIAAWLQTNYGLGAVGLYLAAMAGLTLIAL
          .         *. *    . ..:: .: *  :*.*  .* .:      : ::** :** *

CcoA      VLRPKDTR---
YhjE      LLTHETRHQSL
          :*  :  :
```

AraJ

```
CcoA      MTQTAASAPPLPLRNLIVLVAAQAFLGAQMSMIFTVGGL------AGQSLATNPCLATLP
AraJ      ------------MKKVILSLAL-GTFGLGMAEFGIMGVLTELAHNVGISIPA----AGHM
                      ::::*: :*  . :*  *: :  :* *      .* *: :    *

CcoA      LSLIVLGSVLTAQPMSSFMAVYGRRAGFILATAAGGIGAAISAHALAIGSFPLFCLGSLL
AraJ      ISYYALGVVVGAPIIALFSSRYSLKHILLFLVALCVIGNAMFT---LSSSYLMLAIGRLV
          :*  .** *: *  :: * : *. :  ::: .*   ** *: :     .*: ::.:* *:

CcoA      AGIYMSAQGFYRF---AATDGIAPEHQSKAISWVLAGGLAAAVLGPQLVKLTAQALVVPF
AraJ      SGFPH--GAFFGVGAIVLSKIIKPGKVTAAVAGMVSGMTVANLLGIPLGTYLSQEFS--W
          :*:     .*: .   . :. * * : : *:: :::*  .* :**  * .  :* :   :

CcoA      QATYLAIIAINLAGPLIFAFLRIPAPGRRVKGQAGGRTRGELLRDPVILVAMICGMVSYA
AraJ      RYTFLLIAVFNIAV-MASVYFWVPDIRDEAKG--NLREQFHFLRSPAPWLIFAATMFGNA
          : *:* * .:*:*  :  .:: :*    ..**  . * : .:**.*.  : : . *.. *

CcoA      -LMNLVMTSTPLAVVGCGHTTTNAADIVSAHVLAMYLPSFFTGHLIARFGRETIVGIGLF
AraJ      GVFAWFSYVKPYMMFISGFSETAMTFIMMLVGLGMVLGNMLSGRISGRYSPLRIAAVTDF
           ::  .   .*  :. .*.: *  : *:    *.* * .:::*:: .*:.   *..:  *

CcoA      ILAVAGAVA--LTGVDLEQFFLALMLLGLGWNFGFIGSTAMLA-----------AAHAPE
AraJ      IIVLALLMLFFCGGMKT-----------TSLIFAFICCAGLFALSAPLQILLLQNAKGGE
          *:.:*  :     *:.             .  *.** .:.::*            *:. *

CcoA      ERGTVQGMNDFVVFGGVFLASLSSGGLMTCASADAVAGWQAVNLAMLPFLTLAGAALIWL
AraJ      LLGAAGGQIAFNL-G-SAV-GAYCGGMMLTLG--LAYNYVALPAALLSFAAMSSL-LLYG
            *:. *   * : *   : .  .**:*   .   . .: *:  *:* * :::.  *::

CcoA      VLRPK---DTR-------
AraJ      RYKRQQAADTPVLAKPLG
            : :   **
```

SetC

```
CcoA        MTQTAASAPPLPLRNLIVLVAAQAFLGAQMSMIFTVGGLAGQSLATNPCLAT--------
SetC        MQK-TATTPSKIL----D-LTAAAFL-----LVAFLTGIAGALQ--TPTLSIFLADELKA
            * : :*::*   *      ::* ***     ::  : *:**     .* *:

CcoA        --LPLSLIVLGSVLTAQPMSSFMAVY----GRRAGFILATAA-GGIGAAISAHALAIGSF
SetC        RPIMVGFFFTGSAIMGILVSQFLARHSDKQGDRKLLILLCCLFGVLACT--LFAWNRNYF
              : :.::. **.: .  :*.*:* :    * *  :**  .  * :..:   .*   . *

CcoA        PLFCLGSLLAGIYMSA-QGFYRFAATDGIAP----------EHQSKAISWVLAGGLAAAV
SetC        ILLSTGVLLSSFASTANPQMFALAREHADRTGRETVMFSTFLRAQISLAWVIGPPLAYE-
             *:. * **:.:  :*   :: :*  ..              : . :::**:.  **

CcoA        LGPQLVKLTAQALVVPFQATYLA-IIAINLAGPLIFAFLRIPAPGRRVKGQAGGRTRGEL
SetC        --------L--AMGFSFKVMYLTAAIAFVVCGLIVWLFLPSIQRNIPVVTQPVEILPSTH
                       *: . *:. **:  **: :.* ::: **     .  *  *      .

CcoA        LRDPVILVAMICGMVSYALMNLVMTSTPLAVVGCGHTTTNAADIVSAHVLAMYLPS-FFT
SetC        RKRDTRLLFVVCSMM-WAANNLYMINMPLFIIDELHLTDKLTGEMIGIAAGLEIPMMLIA
             :  . *: ::*.*: :*  ** * . ** ::.  * * : :. : . . .: :*  :::

CcoA        GHLIARFGRETIVGIGL----FILAVAGAVALTGVDLEQFFLALMLLGLGWNFGFIGSTA
SetC        GYYMKRIGKRLLMLIAIVSGMCFYASVLMATTPAVELELQILNAIFLGILCGIGMLYF--
            *: : *:*:. :: *.:     : * .  .:  .*:**  :*  ::**:  .:*::

CcoA        MLAAAHAPEERGTVQGMNDFVVFGGVFLASLSSGGLMTCASADAVAGWQAVNLAM-LPFL
SetC        ---QDLMPEKIGSATTL----------------------YANTSRVGWIIAGSVDGIMVE
                   **: *:.  :                       :  : .**  .. .  : .

CcoA        TLAGAALIWLVLRPKDTR----------
SetC        IWSYHALFWLAIGMLGIAMICLLFIKDI
              :  **:**.:   .
```
