# Supplementary figures and images for: The Escherichia coli MFS-type transporter genes yhjE, ydiM, and yfcJ are required to produce an active bo3 quinol oxidase
Source: PLoS One. 2023 Oct 20;18(10):e0293015. doi: 10.1371/journal.pone.0293015 (PMC10588857; doi:10.1371/journal.pone.0293015)

***cyoA* primers**

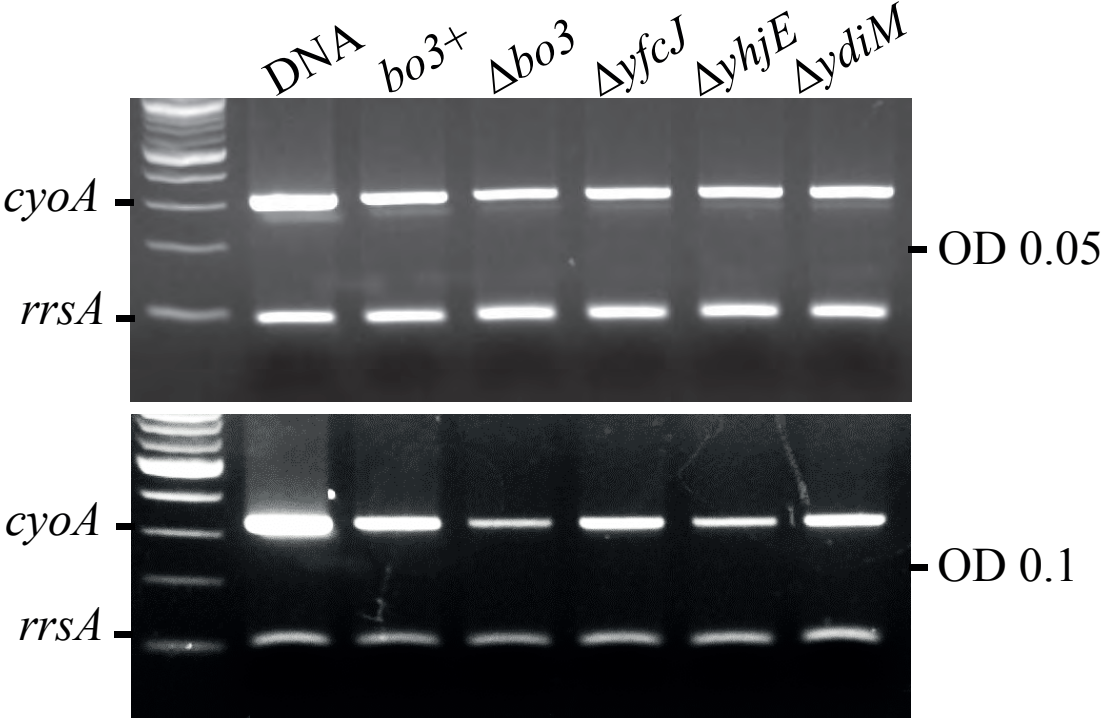

***cyoC* primers**

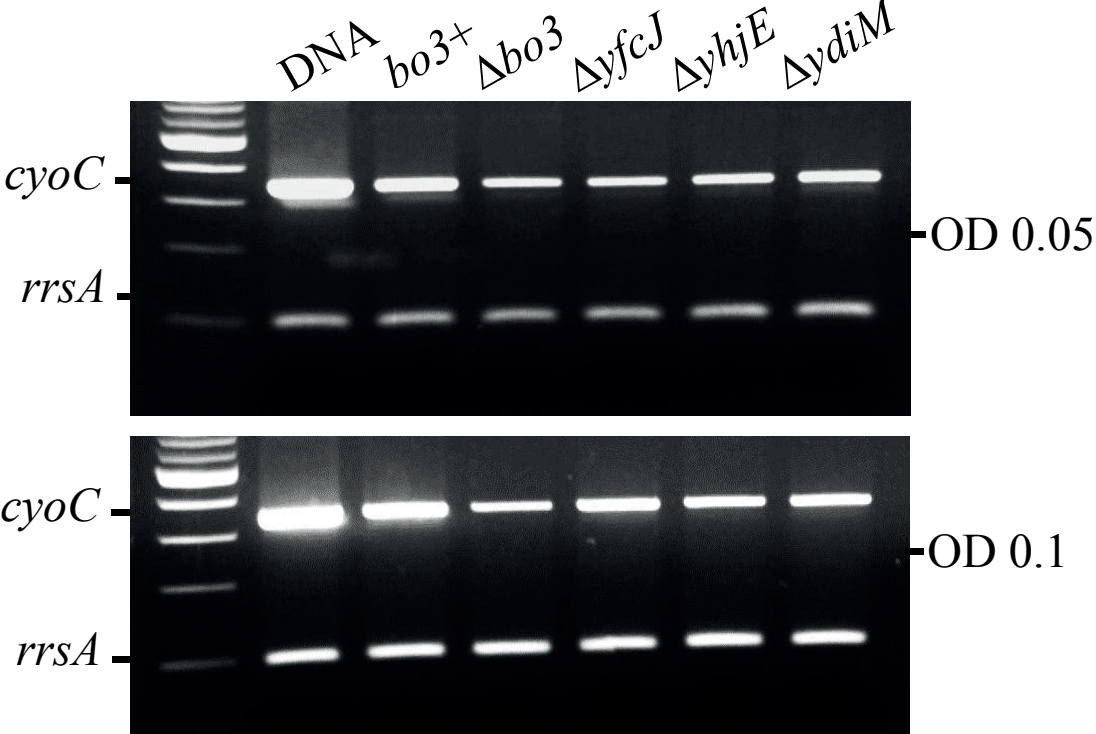

***cyoD* primers**

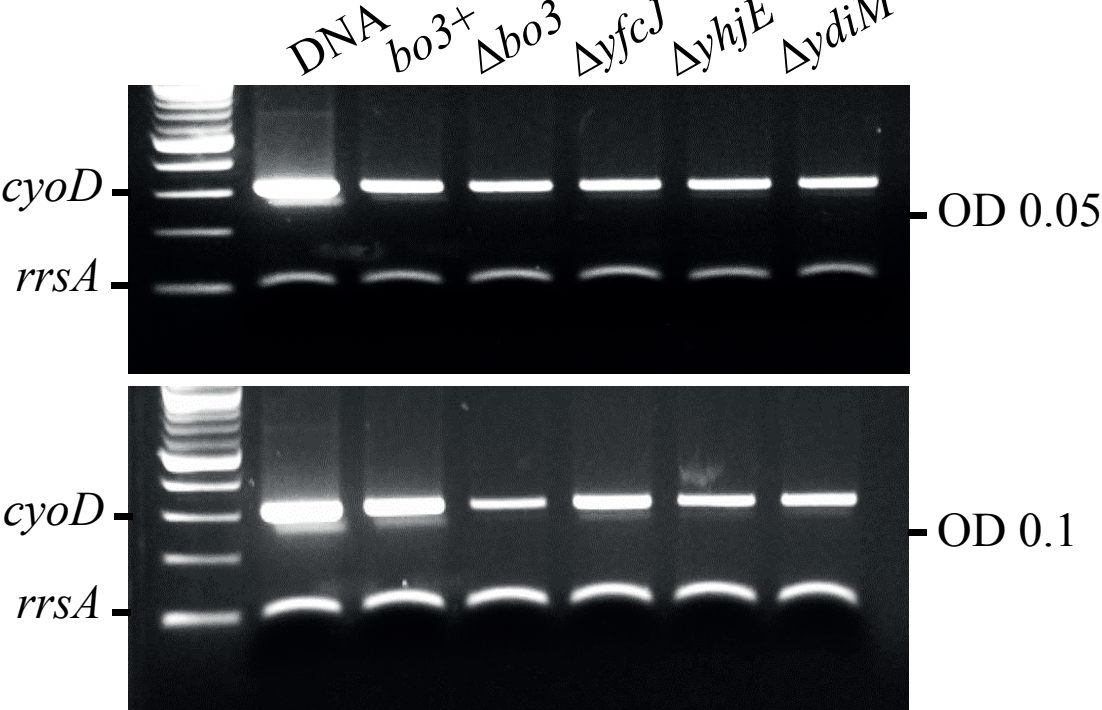

Supplement: S2 Fig — (PDF) [file pone.0293015.s002.pdf]

***cyoB* primers**

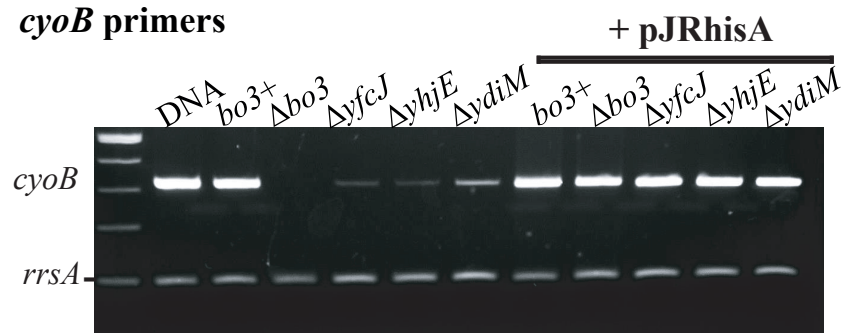

***cyoA* primers**

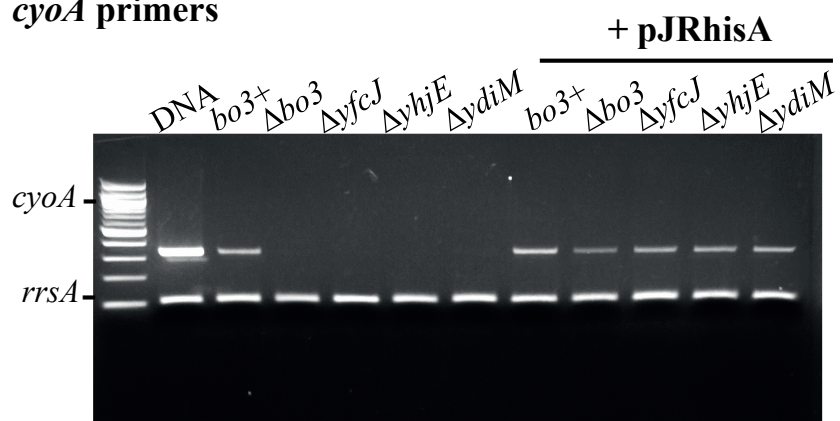

***cyoC* primers**

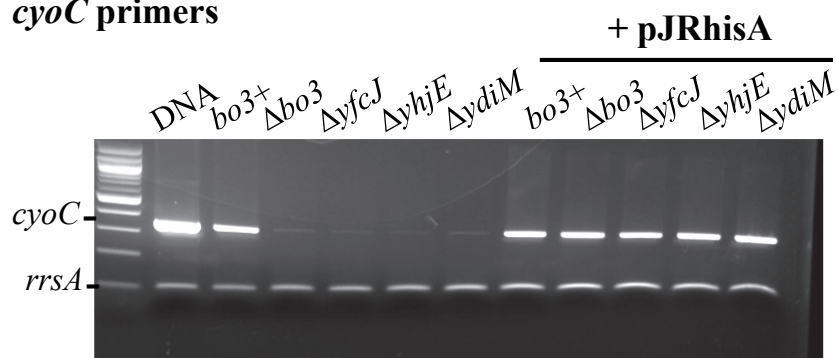

***cyoD* primers**

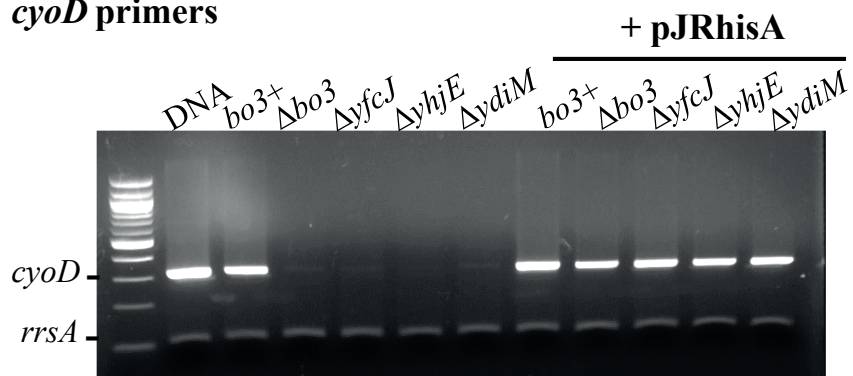

Supplement: S3 Fig — (PDF) [file pone.0293015.s003.pdf]

**A**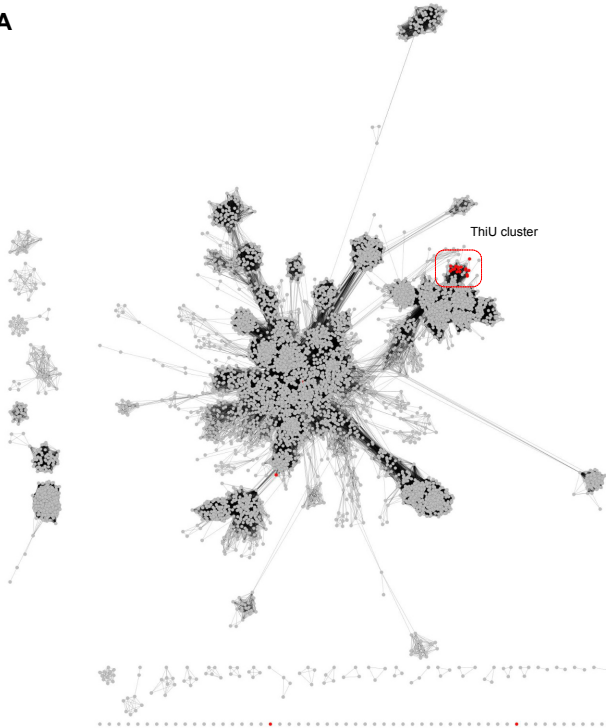**B**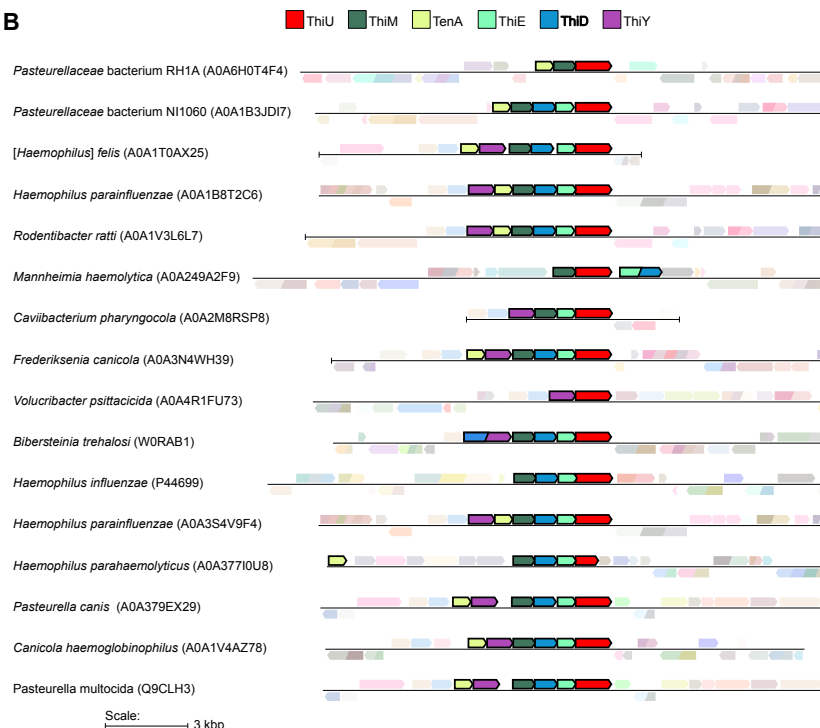

Supplement: S4 Fig — (PDF) [file pone.0293015.s004.pdf]
