# Supplementary material for: The Escherichia coli MFS-type transporter genes yhjE, ydiM, and yfcJ are required to produce an active bo3 quinol oxidase
Source: PLoS One. 2023 Oct 20;18(10):e0293015. doi: 10.1371/journal.pone.0293015 (PMC10588857; doi:10.1371/journal.pone.0293015)

CcoA

-MICGMVSYAL**M**NLV**M**TST--ADIVSA**H**VLA**M**YLPSF-

233 237 261 265

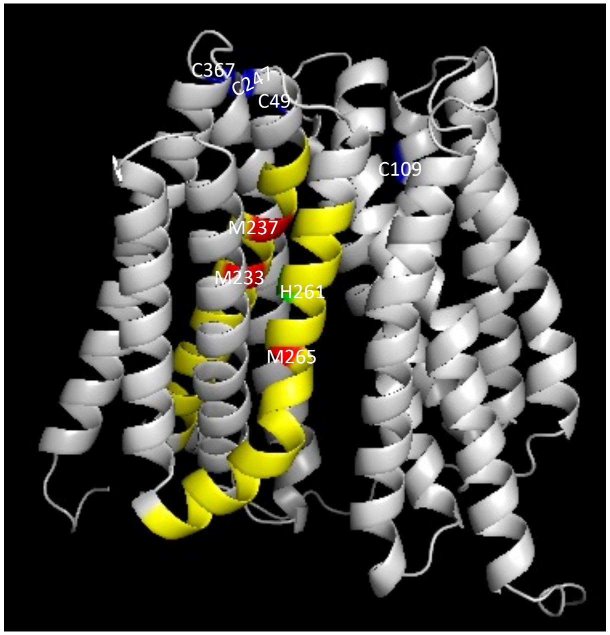

YdiM

-GLYFNYLVHG**M**GVLL**M**SLN**M**ASLE--PFI**M**LG**M**CCY**M**AFFFGIL-

21 26 30 76 79 83

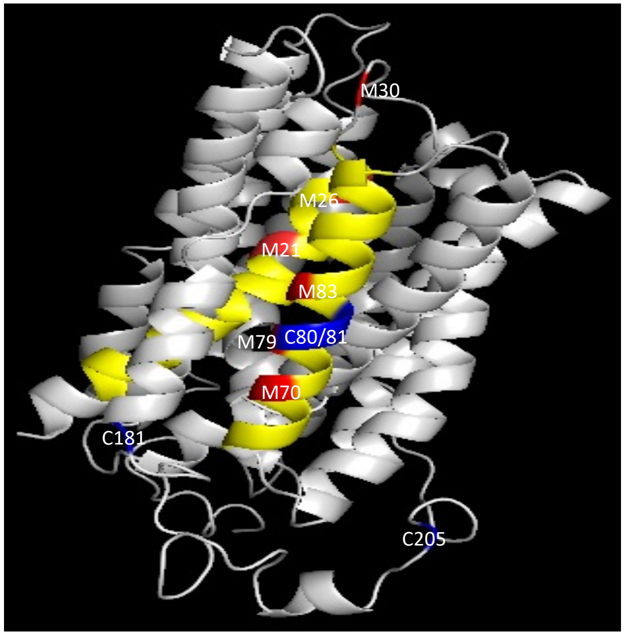

Supplement: S5 Fig — (PDF) [file pone.0293015.s005.pdf]
