# Supplementary material for: The Escherichia coli MFS-type transporter genes yhjE, ydiM, and yfcJ are required to produce an active bo3 quinol oxidase
Source: PLoS One. 2023 Oct 20;18(10):e0293015. doi: 10.1371/journal.pone.0293015 (PMC10588857; doi:10.1371/journal.pone.0293015)
Supplement: S1 File — (DOCX) [file pone.0293015.s006.docx]

**Table S1. Strains and Plasmids**

| **Plasmids or strains** | **Description** | **Antibiotic**  **Resistance** | **Reference** |
| --- | --- | --- | --- |
| ***E. coli strains***  BW25113 | Wild type of *E. coli* K-12 Keio collection: F^-^, DE(*araD*-*araB*)567, *lacZ*4787(del)::*rrnB*-3, LAM^-^, *rph-1*, DE(*rhaD*-*rhaB*)568, *hsdR514* |  | [1] |
| JW0421  JW0723  JW0961  JW5428  JW2319  JW3516  JW5299  JW1588  JW1680  JW3491  JW4072  JW2760  JW2809  JW0386  JW2157  JW3633  *Quinol oxidases*  BF1  BF6  BF13  BF20  **BF24**  **BF17**  *MFS transporters*  BF2  BF3  BF4  BF7  BF8  BF9  BF14  BF15  BF16  **BF21**  **BF22**  **BF23** | ∆*cyoB (bo*_3_ mutant)  ∆*cydB (bd-*I mutant)  ∆*appB (bd-*II mutant)  ∆*ygaY*  ∆*yfcJ*  ∆*yhjX*  ∆*yebQ*  ∆*ynfM*  ∆*ydiM*  ∆*yhjE*  ∆*proP*  ∆*gudP*  ∆*araE*  ∆*araJ*  ∆*setB*  ∆*setC*  ∆*cyoB*::Km^S^  ∆*cyoB*::Km^S^/∆*cydB*::Km^R^  ∆*cyoB*::Km^S^/∆*cydB*::Km^S^  ∆*cydB*::Km^S^  ∆*cydB*::Km^S^/∆*appB*::Km^R^  ∆*cyoB*::Km^S^*/*∆*cydB*::Km^S^/∆*appB*::Km^R^  ∆*yfcJ*::Km^S^  ∆*yhjE*::Km^S^  ∆*ydiM*::Km^S^  ∆*yfcJ*::Km^S^/∆*cydB*::Km^R^ ∆*yhjE*::Km^S^/∆*cydB*::Km^R^  ∆*ydiM*::Km^S^/∆*cydB*::Km^R^  ∆*yfcJ*::Km^S^/∆*cydB*::Km^S^  ∆*yhjE*::Km^S^/∆*cydB*::Km^S^  ∆*ydiM*::Km^S^/∆*cydB*::Km^S^  ∆*yfcJ*::Km^S^*/*∆*cydB*::Km^S^/∆*appB*::Km^R^  ∆*yhjE*::Km^S^*/*∆*cydB*::Km^S^/∆*appB*::Km^R^  ∆*ydiM*::Km^S^*/*∆*cydB*::Km^S^/∆*appB*::Km^R^ | Km^R^  Km^R^  Km^R^  Km^R^  Km^R^  Km^R^  Km^R^  Km^R^  Km^R^  Km^R^  Km^R^  Km^R^  Km^R^  Km^R^  Km^R^  Km^R^  Km^S^  Km^R^  Km^S^  Km^S^  Km^R^  Km^R^      Km^S^  Km^S^  Km^S^  Km^R^  Km^R^  Km^R^  Km^S^  Km^S^  Km^S^  Km^R^  Km^R^  Km^R^ | [1]  [1]  [1]  [1]  [1]  [1]  [1]  [1]  [1]  [1]  [1]  [1]  [1]  [1]  [1]  [1]  This work  This work  This work  This work  This work  This work  This work  This work  This work  This work  This work  This work  This work  This work  This work  This work  This work  This work |
| ***Plasmids***  pJRhisA | pBBr derivative carrying *cyoABCDE* operon | Amp^R^ | [2] |

**Table S2. Primers used for checking the mutants by PCR** (located 100 pb upstream or downstream of the gene of interest) **and for RT-PCR analyses.**

| Primers | Sequence (5’ to 3’) |
| --- | --- |
| CyoB-F  CyoB-R | GACCCAGCCAGAAGGTGAGC  GCAGTCGCTCATCAGGTAGA |
| CydB-F  CydB-R | GTTTGCACGCCTCGGCCCAA  GATTCGACGTGTTCAAGCGC |
| AppB-F3  AppB-R3 | TAATCGCCGAAGTCTACCTG  TTCAGACGCGGGTCCAGCCA |
| SetC-F  SetC-R | TGGTTGTATCAGCGATACTC  GCATACTGTTTTGTCGTTTA |
| AraE-F  AraE-R | GCAGCAATTTAATCCATATT  CAAGGCTGAATAAGGAACGA |
| YhjE-F  YhjE-R | TGCCTGATTTTTGACCATTT  GGCGTGAACGCCTTATCCGG |
| YhjX-F  YhjX-R | TTCTGATGGCATTTCATGCC  GCCAGTTAATTGGCCCAAAT |
| YebQ-F  YebQ-R | CCATCTTTTGCCGGTTAGTT  CCCGCCGCTGGATAAACGAA |
| YdiM-F  YdiM-R | ATTTTTCCACAGAAAGGAAT  ATTGCATGTCATAGTTTGTT |
| YfcJ-F  YfcJ-R | AAAGACGTACGCGTTAAATC  CTTTTCCACCGATGATGGAC |
| YnfM-F  YnfM-R | AACATCTTATTTGAGATTAT  GGAATTGGCTGGCGCTTCGT |
| AraJ-F  AraJ-R | AGGGGCGAATTATCTCTTGG  CCGCGTCTTATCAGGCCTGC |
| GudP-F  GudP-R | CTATAAAAACGGCAAAAAAC  TGCGTTATGTGCCCCACCAA |

**Primers used for RT-PCR analyses.**

| Primers | Sequence (5’ to 3’) |
| --- | --- |
| RrsA-F1  RrsA-R1 | AGGCCTTCGGGTTGTAAAGT  GTTAGCCGGTGCTTCTTCTG |
| CyoBQ-F2  CyoBQ-R3 | TGTCGATTACTGGATATG  AGGCAGGATCAGGATGTA |
| CyoAQ-F  CyoAQ-R | ATCATCATCTTCCTTGCA  GGAGATACCGTCATAAGT |
| CyoCQ-F  CyoCQ-R | ACTGCATTCTGTTCTCTA  GACGTGCAGACCGTGCGT |
| CyoDQ-F  CyoDQ-R | AGTCATTCTACCGATCACA  TGTAGTTGAGGTTCCACAT |

**References for supplemental Table S1.**

1. Baba T, Ara T, Hasegawa M, Takai Y, Okumura Y, Baba M, Datsenko KA, Tomita M, Wanner BL, Mori H **(**2006) Construction of Escherichia coli K-12 in-frame, single-gene knockout mutants: the Keio collection. Mol Syst Biol 2:2006 0008.

2. Rumbley JN, Furlong Nickels E, Gennis RB **(**1997) One-step purification of histidine-tagged cytochrome bo3 from Escherichia coli and demonstration that associated quinone is not required for the structural integrity of the oxidase. Biochim Biophys Acta 1340:131-42.

3. Khalfaoui-Hassani B, Verissimo AF, Koch HG, Daldal F **(**2016) Uncovering the Transmembrane Metal Binding Site of the Novel Bacterial Major Facilitator Superfamily-Type Copper Importer CcoA. mBio 7:e01981-15.

4. Khalfaoui-Hassani B, Trasnea PI, Steimle S, Koch HG, Daldal F (2021) Cysteine Mutants of the Major Facilitator Superfamily-Type Transporter CcoA Provide Insight into Copper Import. mBio 12:e0156721.
