# Supplementary material for: The Escherichia coli MFS-type transporter genes yhjE, ydiM, and yfcJ are required to produce an active bo3 quinol oxidase
Source: PLoS One. 2023 Oct 20;18(10):e0293015. doi: 10.1371/journal.pone.0293015 (PMC10588857; doi:10.1371/journal.pone.0293015)
Supplement: Attachment — Submitted filename: Response to the Reviewers.docx [file pone.0293015.s009.docx]

**Response to the Reviewers**

**Reviewer # 1**

We thank the Reviewer for stating that “the study has been well executed and the results are also well presented”.

*Specific comments*:

1- *The authors should elaborate the introduction with the current literature support.*

The Introduction was elaborated with current literature (ie. recent references), as requested.

2- *The limitations and prospects of the study should be elaborated more for the readers.*

The limitations and prospects of the study were elaborated more for the readers, as requested (see the marked-up copy).

**Reviewer # 2**

We thank the Reviewer for stating that “This research advances our understanding of heme-copper enzyme biogenesis and cellular metabolism. The manuscript is well written and easy to read”.

*Specific comments:*

1. *The paper does not elucidate the specific mechanism by which these MFS-type transporters are involved in bo3-Qox biogenesis. The authors propose that they might play a role in regulating gene expression or stabilizing mRNA transcripts, but this remains speculative. This More detailed mechanistic studies are required to flesh out their specific functions in bo3-Qox production.*

Indeed, elucidation of the specific mechanism (s) (might be more than one…) by which each of these MFS-type transporters is involved in *bo*_3_-Qox biogenesis requires additional extensive studies, which are beyond the scope of this work. Based on the available data, we pointed out the plausible consequences on gene expression and/or assembly of this enzyme.

*2. How are the YhjE, YdiM, and YfcJ transporters involved in copper and iron homeostasis? Is there any evidence suggesting their direct interactions with these metals?*

Whole cell transport data indicate that mutants lacking YhjE and YdiM are affected in copper and iron uptake, respectively. However, no evidence is currently available for direct interaction(s) between these transporters and the metals. Such elaborate experiments require purification of each transporter and specific metal binding assays, with appropriate forms (ie., oxidized/reduced, free/chelated, …) and are beyond this work.

3. *The study suggests that YhjE and YdiM are related to the shikimate pathway. How might their involvement in this pathway be linked to their roles in bo3-Qox biogenesis?*

Currently, it is unclear how the shikimate pathway related to YdiM is linked to *bo*_3_-Qox biogenesis. A possibility is that YdiM might be involved in providing Cu to a shikimate pathway enzyme. Another feature is that the Heme-Copper enzymes contain an unusual Tyr-His covalent linkage at their binuclear metal center that is critical for their activity. Conceivably, changes in the shikimate pathway might be consequential for this issue.

As far as we know, YhjE is not related to the shikimate pathway.

*4. The authors should add “Statistical analyses” in the Materials and Methods. The figure legends should describe the errors bars and N numbers (Ex: Fig. 4)*

As requested, a section describing the Statistical analyses is added to the Materials and Methods section, and p values are added to the figure legends, when appropriate.
