# Supplementary material for: The Escherichia coli MFS-type transporter genes yhjE, ydiM, and yfcJ are required to produce an active bo3 quinol oxidase
Source: PLoS One. 2023 Oct 20;18(10):e0293015. doi: 10.1371/journal.pone.0293015 (PMC10588857; doi:10.1371/journal.pone.0293015)
Supplement: Attachment — Submitted filename: Reviewer comment 08052023.docx [file pone.0293015.s008.docx]

**Journal:** PLOS ONE

**Manuscript Type:** Research Article

**Manuscript ID:** PONE-D-23-23379

**Title:** The *Escherichia coli* MFS-type transporter genes yhjE, ydiM, and yfcJ are required to

produce an active bo3 quinol oxidase

The current work by Khalfaoui-Hassani *et al* investigates molecular insights into the production of an active bo3 quinol oxidase in *Escherichia coli*. The group identified a subset of uncharacterized MFS transporters, based on the presence of putative metal-binding residues, as likely candidates for the missing Cu transporter. Phylogenomic analyses also suggest plausible roles for the YhjE, YdiM, and YfcJ transporters, and overall findings illustrate the diverse roles that the MFS-type transporters play in cellular metal homeostasis and production of active heme-Cu oxygen reductases. Overall evaluation shows that the study has been well executed and the results are also well presented. The conclusion of the study is substantiated by the experimental outcomes. The authors have listed the limitations of their study with the future scope/directions. There are a few minor suggestions. If rectified, the article will be suitable for publication in PlosOne.

1. The authors should elaborate the introduction with the current literature support.
2. The limitations and prospects of the study should be elaborated more for the readers.
